# Recent Advances in miRNA Delivery Systems

**DOI:** 10.3390/mps4010010

**Published:** 2021-01-20

**Authors:** Ishani Dasgupta, Anushila Chatterjee

**Affiliations:** 1 Horae Gene Therapy Center, Department of Pediatrics, University of Massachusetts Medical School, Worcester, MA 01605, USA; ishani.dasgupta@umassmed.edu; 2 Department of Immunology and Microbiology, University of Colorado School of Medicine, Aurora, CO 80045, USA

**Keywords:** miRNA, miRNA delivery, miRNA therapeutics, viral vectors, non-viral vectors

## Abstract

MicroRNAs (miRNAs) represent a family of short non-coding regulatory RNA molecules that are produced in a tissue and time-specific manner to orchestrate gene expression post-transcription. MiRNAs hybridize to target mRNA(s) to induce translation repression or mRNA degradation. Functional studies have demonstrated that miRNAs are engaged in virtually every physiological process and, consequently, miRNA dysregulations have been linked to multiple human pathologies. Thus, miRNA mimics and anti-miRNAs that restore miRNA expression or downregulate aberrantly expressed miRNAs, respectively, are highly sought-after therapeutic strategies for effective manipulation of miRNA levels. In this regard, carrier vehicles that facilitate proficient and safe delivery of miRNA-based therapeutics are fundamental to the clinical success of these pharmaceuticals. Here, we highlight the strengths and weaknesses of current state-of-the-art viral and non-viral miRNA delivery systems and provide perspective on how these tools can be exploited to improve the outcomes of miRNA-based therapeutics.

## 1. Introduction

The spatiotemporal expression of microRNAs (miRNAs) in eukaryotes, a class of small single-stranded non-coding RNAs (18–25 nucleotides), plays a critical role in post-transcriptional gene regulation [1]. MiRNAs serve as modulators of gene expression by annealing to complementary sequences in the 3′ or 5′ untranslated regions (3′UTR or 5′UTR) of target mRNAs to block translation machinery and drive mRNA cleavage [2,3,4,5]. Currently, 4571 human miRNAs (1917 precursors, 2654 mature) are annotated in public repositories [6], and it is estimated that these non-coding RNAs regulate >30% of protein-coding genes involved in different biological processes [7,8]. Additionally, the availability of the human miRNA tissue atlas, which provides a comprehensive catalogue of tissue-specific miRNA distribution and expression patterns, enables investigators to probe the physiological and pathological contributions of different miRNAs [9,10]. Several reports implicate that dysregulated or dysfunctional miRNAs are associated with diverse human pathologies, including cancer, cardiovascular, neurodegenerative, inflammatory, genetic, and infectious diseases [11,12,13,14,15,16,17].

With the emerging evidence that miRNAs are involved in the onset and progression of diverse biological anomalies, there has been a drastic surge of interest in miRNA-based therapies over the last few decades [18,19]. Therapeutic approaches have been developed to either suppress or restore the expression of disease-associated miRNAs (Table 1). In circumstances where reduced miRNA expression drives the disease, miRNA mimics can be used to restore their expression and function [19,20,21,22]. In contrast, anti-miRNAs (antagomirs) are exploited to counteract the activity of upregulated miRNAs responsible for disease [22,23,24]. However, the safe and efficient delivery of miRNA mimics or antagomirs to target tissues remains a significant challenge for miRNA-based therapies. Major limitations associated with miRNA delivery are susceptibility to degradation by nucleases, rapid clearance from blood, immunotoxicity, and low tissue permeability [25,26,27,28,29]. Chemical modifications of miRNAs have significantly improved their stability and provided protection against nucleases [30,31,32,33]. Further, several oligonucleotide carriers have been developed to enhance stability and improve tissue penetration. In vivo viral and non-viral delivery miRNA methods, the challenges associated with the delivery methods, and strategies to circumvent them for a multitude of diseases, with a focus on cancer therapy, have been extensively reviewed [34,35,36]. These reviews also provide a detailed discussion on the miRNA expression profiles implicated in cancers. From this perspective, we emphasize the delivery aspects of miRNA in various human diseases and draw attention to some newly evolving miRNA delivery techniques that have not been covered in the recent reviews. Here, we provide a holistic overview of the viral and non-viral delivery systems developed to maximize miRNA therapeutic efficacy, highlight selected examples of their applications in various human diseases, comment on current clinical trials in the field, and offer perspectives on the future design and development of miRNA delivery technologies.

## 2. Virus-Based miRNA and Anti-miRNA Oligonucleotide Delivery Systems

**Figure 1:**
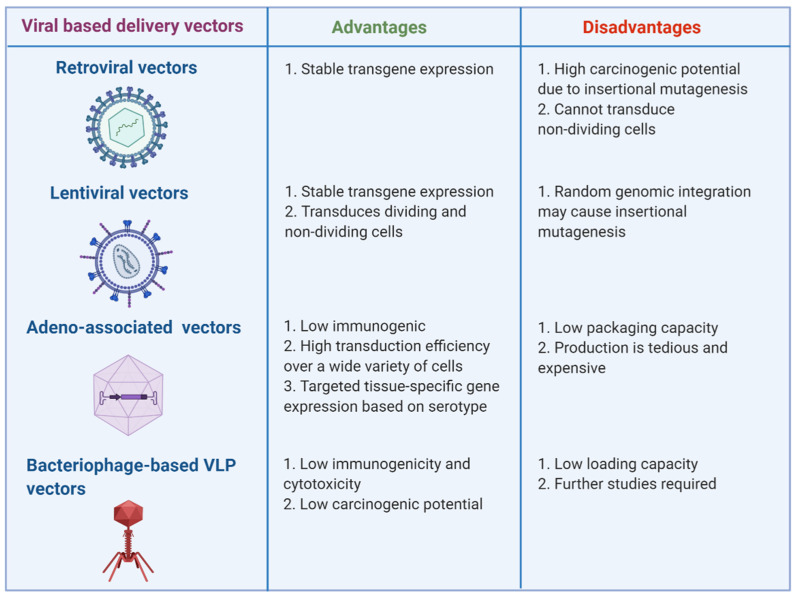
Key advantages and disadvantages of virus-based vectors used for miRNA delivery are highlighted. Viral vectors represented here include retroviral, lentiviral, adeno-associated, and bacteriophage-based VLP vectors. Figure was created with BioRender.com.

Genetically modified viruses can efficiently transfer desired oligonucleotides into different tissue types and drive elevated levels of gene expression for protracted periods. In the context of eukaryotic viruses, the pathogenic determinants are eliminated from the viral genome to reduce toxicity and accommodate the transgene(s). Over the past few decades, a variety of viral delivery vehicles have emerged that can be adapted for specific transgenes, treatment purposes, and targeted cell types. Here, we identify distinct characteristics and limitations of major virus-based vectors used for miRNA or anti-mRNA (also known as antagomir) delivery, including retroviral, lentiviral, adenoviral and adeno-associated virus (AAV) (reviewed in [59,60]), and bacteriophage-based virus-like particle (VLP) vectors (Figure 1) [61,62]. 

### 2.1. Retroviral and Lentiviral Vectors

Retroviral vectors (RVs), developed from lipid-enveloped RNA viruses, have been pivotal for the stable transfer of therapeutic genes into dividing cells [60]. Most RVs are derived from the Moloney murine leukemia viruses (MoMLVs) that have a relatively simple genome structure encoding the Gag, Pol, and Env proteins flanked by long terminal repeats (LTR) [63]. Upon recognition and binding to specific cell surface-associated receptors, viral RNA enters the cytoplasm, is reverse transcribed into dsDNA, and proceeds to randomly integrate into one of the host chromosomes. The ability to integrate exogenous DNA into the host chromosome imparts a “Janus-faced” character to RVs. While genomic integration accentuates persistent transgene expression, insertional inactivation of critical genes or their regulatory elements can be detrimental for the cell [64,65]. Nevertheless, RV-mediated miRNA delivery has been shown to be promising in regenerative medicine. For example, heightened expression of miR-138 in murine embryonic fibroblasts led to the downregulation of the p53 signaling pathway and consequently favored induced pluripotent stem (iPS) cell production, which has implications in regenerative medicine [66]. 

Members of the lentivirus genus of *Retroviridae* family, including immunodeficiency viruses of bovine (BIV), feline (FIV), equine (EIAV), simian (SIV), and human (HIV-2), have been tailored to develop lentiviral vectors (LVs) [67,68,69,70,71]. In contrast to RVs that can only access the host chromosome when the nuclear membrane is dismantled during mitosis, LVs can actively translocate across an intact nuclear membrane via the nuclear pores and, thereby, can target both quiescent and non-quiescent cells [72]. Another major hurdle associated with RVs is the significant risk of developing oncogenesis as a consequence of insertional mutagenesis [73,74]. Because LVs preferably integrate within actively transcribing units, they have reduced likelihood of triggering insertional oncogenesis [75,76,77]. Several studies have effectively used LVs for the delivery of therapeutic miRNA mimics or antagonists. In a mouse model of chronic lymphocytic leukemia (CLL), lentiviral delivery and subsequent elevated levels of microRNAs, miR-15a, and miR-16 caused the depletion of malignant B cells and mitigated the disease [78]. Another study explored the therapeutic potential of lentiviral miR-494 sponge and demonstrated that these anti-miRNAs could sequester miR-494 molecules away from their cellular targets to reduce tumor growth and metastasis [79].

### 2.2. Adenovirus and Adeno-Associated Virus Vectors

Adenoviruses (Ad) and adeno-associated viruses (AAV) are engineered from non-enveloped viruses with double-stranded and single-stranded DNA genomes, respectively. AAVs emerged as potent gene delivery systems owing to their non-pathogenic profile, broad target tissue spectrum, and sustained presence in the system [80]. Additionally, two key features of these viruses contribute to their therapeutic success. Similar to LVs, both Ads and AAVs can infect resting or dividing cells. However, unlike RVs and LVs, these viruses do not integrate into the host chromosome and hence are unlikely agents of insertional oncogenic activation. Compared to RVs and LVs that can carry up to 8 Kb of foreign nucleotide sequences, Ads can carry as much as 38 kb of alien DNA [60]. Although AAVs have a fairly limited capacity for exogenous DNA (~4.8 Kb), they have sufficient room to accommodate most miRNA cassettes [81]. Several DNA viral platforms have been designed to deliver miRNAs (Table 1). Further, Miyazaki et al. reported that AAV vector-mediated delivery of miR-196a can silence Elav-like family member 2 (CELF2) and subsequently reduce androgen receptor (AR) mRNA stability, leading to the attenuation of spinal and bulbar muscular atrophy (SBMA) phenotypes [82]. Recently, Tang et al. found that recombinant adenovirus-delivered hemagglutinin-specific artificial miRNAs could provide protection from lethal strains of influenza virus and mitigate disease manifestations [83]. In another study, Pourshafie et al. used an AAV delivery system with high transduction efficiency to overexpress miR-298 and attenuated neuromuscular diseases in mice models [40]. Despite these successes, studies in large animal and human patients noted immune activation against AAV [84]. Indeed, several parameters, including specific properties of the transgene, vector dose and serotype, administration route, host species, and the presence of pre-existing neutralizing antibodies, may influence the development of an immunological response against AAV [84]. Because the promoter and kinetics of transgene expression strongly affect the immune response elicited to AAV, efforts have been made to achieve focused transgene expression using highly compact tissue-specific promoters and enhancers [85]. In this context, investigators have incorporated tissue-specific miRNA target sequences into the 3′-UTR of an AAV vector cassette to prevent unintentional transgene expression in the liver without disrupting the transgene expression in other tissues [86].

### 2.3. Bacteriophage-Based VLP Vectors

The success of eukaryotic virus-based miRNA delivery systems can be attributed to their high transduction efficiency, broad tropism, and long-term expression. However, the potency of these delivery vehicles is frequently restricted by their high cytotoxicity [87], carcinogenic potential [88], and strong immunogenicity [89]. To circumvent these challenges, researchers have exploited the encapsidation system of viruses that infect bacteria, termed bacteriophages, to deliver miRNAs. Pan et al. used *Escherichia coli* as cellular factories to package miRNAs in capsids of bacteriophage MS2 and subsequently cross-linked the miRNA carrying VLPs with cell-penetrating peptides (CPP) to achieve efficient transduction [61]. Succeeding studies have shown that MS2 VLP-based miRNA delivery systems containing disease-specific miRNAs could be harnessed to treat a chronic autoimmune disease, osteoclastogenesis, and hepatocellular carcinoma [90,91,92]. Another study has demonstrated that targeted delivery of miRNA-23b via bacteriophage PP7 VLPs to hepatoma cells can inhibit the migration of these cells and potentially reduce the risks of various associated cancers [62]. Another group has used folate-conjugated phage packaging RNA (pRNA) as a vehicle to deliver artificial miRNAs targeting the 3′UTR of coxsackievirus B3 (CVB3) strains Kandolf and CG, a common cause of myocarditis [93]. The pRNA is a 117-nucleotide-long RNA molecule found in *Bacillus subtilis* bacteriophage phi 29 that is essential for phage DNA encapsidation [94]. The unique structural features of pRNA enable the formation of oligomeric assemblies, and consequently, pRNAs have the ability to carry both therapeutic molecules and targeting ligands for efficient drug delivery [95]. Overall, research on phage-derived miRNA delivery systems is still in its early stages, and future studies evaluating the immunogenicity profile and pharmaceutical production of these vehicles will be imperative for the clinical exploitation of phage-derived vehicles for miRNA delivery.

## 3. Non-Viral-Based miRNA and Anti-miRNA Oligonucleotide Delivery Systems

**Figure 2:**
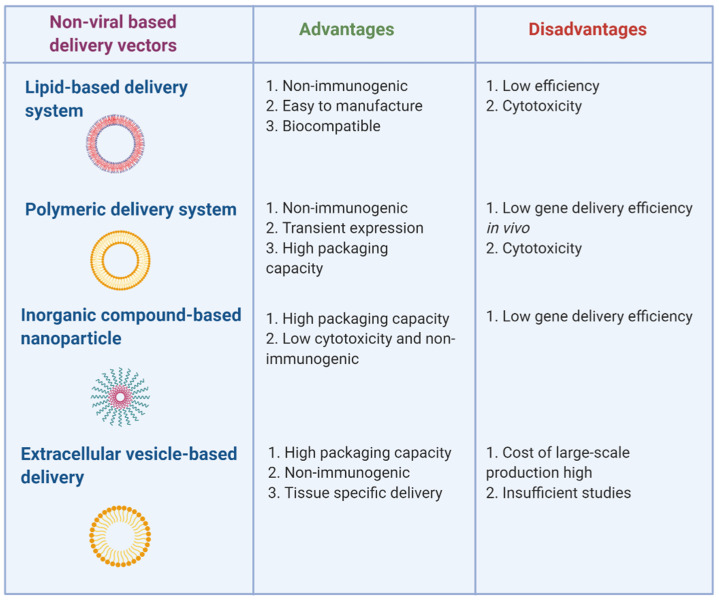
Strengths and weaknesses of non-viral miRNA delivery technologies. Lipid-, polymeric-, inorganic-, and exosome-based delivery methods are shown. Figure was created with BioRender.com.

Despite the highly efficient viral-based miRNA delivery systems, they are associated with high immunogenicity, toxicity, and size limitation. To overcome these challenges, less toxic and biocompatible non-viral-based miRNA delivery approaches have come to light. The non-viral delivery systems ensure successful delivery of miRNA or miRNA-expressing vectors inside the cell without being subjected to nuclease degradation. Here, we discuss the different chemical methods of non-viral miRNA delivery, including lipid, polymer, inorganic, and extra-cellular vesicle carrier-based approaches (Figure 2).

### 3.1. Lipid-Based Delivery Systems

Lipid-based nanocarriers are the most widely used non-viral delivery methods [96]. Primarily, cationic lipids with hydrophilic heads and hydrophobic tails form a complex with the anionic nucleic acid, resulting in a lipoplex [97,98]. These cationic lipoplexes have a high affinity with the cell membrane, and they are non-immunogenic and easy to manufacture. Many commercially available cationic lipoplexes—for example, Lipofectamine^®^ RNAi-MAX, SiPORT™ (Invitrogen) [99,100], SilentFect™ (Bio-Rad) [101], and DharmaFECT^®^(Dharmacon) [102]—have been routinely used for miRNA delivery. Although cationic liposomes have been used to deliver miRNA in vivo, the efficiency is low. Several modifications have been employed to circumvent this problem. Conjugating a polyethylene glycol (PEG) functional group to the cationic lipids helps in evading phagocytosis, thereby improving the overall efficiency [103]. A study reported that PEG-fused liposomes enabled successful miR-126 delivery, resulting in enhanced blood flow and angiogenesis in a hindlimb ischemia model [104]. Several studies have shown the successful in vivo transport of lipoplexes, including the systemic delivery of miR-29b fused with DOTMA, cholesterol, and PEG in non-small-cell lung cancer (NSCLC) cells [46] and miR-34a delivery mediated by lipid nanoparticles, consisting of cholesterol, DDAB [105]. Another comparable cationic lipoplex mixture containing dimethyldioctadecyl ammonium bromide (DDAB), cholesterol, and vitamin E TPGS transported pre-miR-107 to head and neck squamous cell carcinoma (HNSCC) cells and greatly alleviated the tumorigenesis of HNSCC in vitro and in vivo [106]. Cationic DOTAP enabled the co-delivery of doxorubicin and miR-101 in hepatocellular carcinoma (HCC) cells and also yielded desirable results [107]. Another successful combination using cationic liposome nanocarriers has been developed for treating melanoma [108]. These nanoparticles were successful in delivering paclitaxel and Bcl-2 siRNA for treating melanoma synergistically. Besides cancer, cationic lipoplexes containing anti-miR-712 were able to treat atherosclerosis in inflamed endothelial cells [44]. A major disadvantage of these cationic lipoplexes is their non-specific interactions with other undesirable proteins, leading to adverse effects and their instability. This issue has been alleviated by the recent use of neutral liposomes for miRNA delivery. Systemic administration of miRNA-34a delivered by a neutral liposome emulsion in a NSCLC mouse model yielded even distribution in desired tissues and a subsequent reduction in tumor size. Neutral DOPC (1,2-dioleoyl-*sn*-glycero-3-phosphatidylcholine) liposomes were able to deliver miRNA-506 mimics or miRNA-520 in an ovarian cancer orthotopic mouse model, leading to significant tumor suppression [109,110]. Another example of DOPC liposomes complexed with miR-2000 proved effective in inhibiting tumor growth in orthotopic lung cancer [111].

One of the concerns in liposome-based delivery may be the non-specific or systemic accumulation of the miRNA. Several approaches have been employed to enhance targeted miRNA delivery to specific cells or tissues. Using targeting ligands in the liposome formulations that can bind specifically to receptors on the target cell enables tissue-specific delivery. Generally, transferrin and folic acid are widely used ligands for targeting cancer cell receptors. Antibodies against matrix metalloproteases (MMP), vascular endothelial growth factor (VEGF), vascular cell adhesion molecule-1 (VCAM), and integrins fused to lipid nanoparticles can be used to specifically target cancer cells of interest [112]. A more recent strategy employs the use of aptamers that bind desired cell surface receptors for delivering miRNA or siRNA lipid nanoparticles [113].

### 3.2. Polymeric Delivery Systems

Polymeric delivery methods primarily use polyethyleneimines (PEIs), wherein the positively charged amine groups form a complex with the anionic RNA, thereby shielding the RNA from being degraded and enabling cellular uptake [114]. Both low- and high-molecular-weight linear and branched PEIs have been utilized as miRNA carrier systems [115]. Comparatively, low-molecular-weight PEIs are less cytotoxic and were shown to effectively deliver miR-33a mimics and miR-145 into colon cancer xenograft mice, resulting in decreased tumor growth [49]. However, low transfection efficiency and cytotoxicity render PEIs unfavorable for clinical applications. Other polymers, such as PEG or poly L-Lysine (PLL), when covalently fused to PEI, help in improving its biocompatibility, thereby making it less toxic to cells [116]. PEG/PEI nanocomplex polymeric vectors proved to be stable and enabled effective miR-150 transfection in human leukemia cells [117]. A copolymer of poly lactic acid (PLA) and poly glycolic acid, namely poly lactide-co-glycolide (PGLA), is an FDA-approved biodegradable polyester implicated in anti-miRNA delivery [118]. The hydrophobicity of PGLA impairs its miRNA delivery efficacy. Positively charged synthetic polyadenoamine (PAMAM) dendrimers are biodegradable and have higher transfection efficiency and lower cytotoxicity compared to other polymers. An intravenous injection of PAMAM dendrimers and PEG-nanographene oxide (NGO) linked to anti-miR-21 was successfully delivered to target tumor tissues in a recent study [119]. Another approach that has been employed is the use of polymeric micelles, consisting of a hydrophilic and a hydrophobic polymer. Doxorubicin and tumor suppressor miR-34a were co-delivered to cancer cells using this polymeric micelle strategy [120]. In addition to these synthetic polymers, less toxic, natural cell-penetrating peptides (CPPs) are also involved in miRNA delivery. CPP from naturally occurring protamine acted as a carrier for miR-29b transfer to osteogenic stem cells [121]. Chitosan is another example of a biocompatible, natural polysaccharide and its galactosylated form drives miRNA-16 precursor transport to mouse colonic macrophages [122,123,124].

### 3.3. Inorganic Compound-Based Delivery Systems

Inorganic compounds that are implicated in miRNA delivery primarily include gold [125], Fe_3_O_4_-based [126], and silica-based nanoparticles [127]. These nanoparticles, when fused to a functional thiol or amino groups, can ensure stronger interaction with the cargo (miRNA), thereby facilitating its delivery [125]. Administration of thiol-modified anti-miR-155 gold nanoparticles helped to restore cardiac function in a diabetic mouse model [128]. Moreover, gold nanoparticles conjugated to PEG led to the successful delivery of miR-1 cancer cells, associated with high transfection efficiency and low cytotoxicity [129]. Other examples include anti-miRNA-155 embedded in silica nanoparticles that form a complex with dopamine and AS1411 aptamer resulted in tumor growth inhibition in colorectal cancer [130]. Silica nanoparticles are thermostable, biocompatible, and have large surface area and pore volume, making them favorable miRNA and anti-miRNA vehicles [131]. A nanocomplex, consisting of Fe_3_O_4_ nanoparticles and polymers, namely polyglutamic acid and PEI, showed promising results by delivering miR-100 in vivo. In patient xenografts, systemic injection of this nanocomplex in combination with the routine docetaxel chemotherapy suppressed tumor growth, thereby improving its therapeutic potential [132]. 

### 3.4. Extracellular Vesicle-Based Delivery Systems

Extracellular vesicles (EVs) are heterogenous membrane vesicles involved in intercellular communication, enabling transport of biomolecules, such as proteins, miRNA, etc., via the bloodstream [133]. The presence of the CD47 marker on their surface protects them from phagocytic clearance. Additionally, surface modification of EVs facilitates targeted biomolecule delivery to specific tissues. These features render them promising miRNA delivery vehicles [134]. Depending upon their biogenesis, EVs are classified into exosomes, microvesicles, and apoptotic bodies. Exosomes (40–120 nm in diameter), primarily formed from late endosomes, have been used as effective carriers of miRNA [135,136]. The low cytotoxicity and antigenicity of exosome-based delivery makes it highly efficient. To enrich exosomes with miRNAs, two strategies have been employed. A cell line overexpressing the miRNA of interest is generated, resulting in increased miRNA expression and exosome secretion with the encapsulated miRNA. Another strategy is isolating exosomes and then enriching them with miRNAs. Enrichment of exosomes with miRNA is commonly achieved by transfecting adipose tissue-derived stem cells and mesenchymal stem cells with the miRNA of choice. The potential of the EVs as carriers of exogenous therapeutic miRNA has been discussed in detail in earlier reports [137]. MiRNA-enriched exosomes have been used in a wide variety of diseases, including brain disorders [138,139,140,141], cardiac diseases [142,143,144], muscular disorders [126,145], cancer [146,147] etc. Exosome-mediated delivery of miR-193b helped to diminish amyloid precursor protein levels, in an attempt to ameliorate Alzheimer’s disease. Synaptic transmission in astrocytes is enhanced by miR-124a secretion via extracellular vesicles that regulate the glutamate transporter [148]. In myocardial infarction disease models, intravenous injection of miRNA-126-enriched exosomes helped to ameliorate cardiac injury and fibrosis [144]. Additionally, miRNA-126–3p and 5p successfully delivered by exosomes from endothelial progenitor cells helped to regulate vascular permeability in cecal ligation and puncture (CLP)-triggered sepsis [149]. MSC-derived exosomes that deliver miR-92-a-3p suppress cartilage degeneration and can be used as potential osteoarthritis treatment [145]. In another study, bone marrow MSC-derived exosomes enriched with anti-miRNA-375 were used to restrict apoptosis during islet transplantation in humanized mice [150]. In a recent study, exosomes were engineered to co-deliver an anticancer drug along with miR-21 inhibitor in colorectal cancer cell lines to circumvent drug resistance and improve the efficacy of cancer treatment. The ability of exosomes to regulate immune system makes them an attractive tool for miRNA delivery in autoimmune diseases [151,152]. The levels of circulating exosomes are high in SLE, rendering them novel biomarkers of SLE progression [153]. Further advancements in exosome-based miRNA delivery will prove beneficial for future clinical implications in SLE. Besides exosomes, other EVs such as microvesicles and apoptotic bodies also function as miRNA carriers. A study reported microvesicles enriched with miRNA-29a/c that were able to suppress tumor development in gastric cancer [154]. Endothelial cell-derived apoptotic bodies containing miR-126 induced CXCL12 secretion, thereby protecting mice against atherosclerosis [155]. 

EVs enriched with the exogenous therapeutic miRNA have been used as efficient delivery vehicles and their applications in cell-based delivery are rapidly emerging. Despite their efficacy, the mass production of EVs remains a challenge. Further characterization of the EVs, including regulation of their biogenesis, determining the source from which they have been derived, and the route of administration, needs to be carried out to achieve large-scale production on a clinical scale. Additionally, thorough immune profiling needs to be conducted post exosome delivery to evaluate the recipient’s immune responses, thereby determining the clinical feasibility of this method. Thus, advancements in the isolation of EVs on a commercial scale, strategies to enhance miRNA loading on EVs, and safe delivery to target tissues are exciting avenues that need further exploration. 

### 3.5. Emerging Methods of miRNA Delivery Systems

As miRNA-based therapy is growing in popularity as a means for treating diverse human diseases, novel oligonucleotide delivery strategies are being investigated to enhance the treatment outcomes. Gasparello and colleagues found that argininocalix[4]arene 1, a new synthetic cationic surfactant with basic amino acids clustered on a rigid macrocyclic scaffold, can efficiently transfer miRNAs and anti-miRNA molecules to target cells in vitro [156]. Another new multivalent macrocyclic carrier, tetraargininocalix[4]arene (1), has been effectively used as a non-covalent vector for a peptide nucleic acid–anti-miR nanocomplex [157]. Although such novel macrocyclic carriers showed high transfection efficiency and low cytotoxicity in a variety of cell lines, in vivo validation of these characteristics will be critical for the development of therapeutic protocols.

The low transfection efficiency of neutrons and the presence of the blood–brain barrier, which prevents the delivery of miRNA-based therapeutics to the central nervous system, present significant obstacles to the use of oligonucleotide-based therapies in the brain. In this context, Soto-Sánchez et al. first demonstrated that a polymeric magnetic particle, termed Neuromag^®^, could be employed to deliver nucleic acids to pyramidal cells in the rat visual cortex [158]. In a recent study, investigators demonstrated the efficacy of Neuromag^®^-complexed anti-miR-134 for silencing miR-134, a miRNA implicated in excitatory neurotransmission, neuritogenesis, spinal growth, and neuroplasticity [159].

To overcome the restricted efficiency and specificity of non-viral oligonucleotide carriers, researchers have engineered a nanobody-functionalized nucleic acid nanogel for the targeted delivery of miRNAs to tumor cells and to prevent tumor growth [160]. In another work, researchers engineered a multipronged DNA star motif that can carry three miRNA molecules and form a Shuriken-like shape upon miRNA loading [161]. In this proof-of-concept study, Qian et al. demonstrated that the DNA Shuriken nanostructure could be used to deliver a tumor suppressive miRNA to human colorectal cancer cells [161]. Nahar et al. assembled a DNA nanostructure carrying multiple anti-miR overhangs for the synergistic repression of multiple oncomiRs and prevented cell cycle progression in cancer cells [162]. Together, these studies demonstrate that the programmability of DNA nanostructures holds great promise to further explore the delivery of miRNA-based therapeutics.

## 4. Conclusions

Concerted efforts from academic research laboratories and pharmaceutical companies bolstered the progress of miRNA-based drug candidates to clinical trials for the treatment of diverse pathologies, ranging from kidney diseases to cardiac abnormalities, from different types of cancer to infectious diseases (Table 2 and reviewed in [7,163,164]). Currently, more miRNA-based therapeutics are in the pre-clinical stage or in the development pipeline for treating post-myocardial infarction remodeling, vascular disease, cardiac fibrosis, abnormal red blood cell production such as polycythemia vera, cardiometabolic disease, peripheral arterial disease, and chronic heart failure [164]. Despite these provocative advances, miRNA drug candidates are yet to reach phase III clinical trial and receive clearance from the US Food and Drug Administration (FDA) for medical intervention. Successful translation of miRNA-based strategies from bench to bedside remains dependent on the development of miRNA delivery vehicles that couple essential features such as high loading capacity, stability, enhanced half-life in circulation, minimal toxicity, and prevent the rapid degradation of their cargo.

Although several viral and non-viral miRNA delivery systems have been successfully used in vivo, all of these approaches have pros and cons (Figure 1 and Figure 2). While non-viral vectors are safe, they have low delivery efficiency. In contrast, viral vectors have higher transfection efficiency, but face the challenges of being immunogenic and cytotoxic. Chemical modifications and conjugations are being designed to alleviate toxicity and optimize transfection efficiency. For example, the half-life of lipid nanoparticles in sera was greatly increased by the conjugation of the lipids with hydrophilic and flexible polyethylene glycol (PEG) [165]. The potency of PAMAM has been improved through PEGylation whereas that of PEI was enhanced by generating disulfide cross-linked low-molecular-weight PEI that are assembled with biodetachable anionic groups [163]. Combined delivery of miRNAs and drugs is also being explored to augment therapeutic efficacy. For instance, biocompatible silica-based nanostructures have been employed to co-deliver anti-miR-221 and Temozolomide (TMZ) for treating drug-resistant glioma cells [166].

Most in vivo administration of miRNA-based therapeutics relies on systemic injection, which is expensive, has low efficacy, and can lead to adverse side effects. Therefore, targeted miRNA delivery platforms that improve the homing of delivery vehicles to specific tissues are being explored. Active targeting has been achieved by tethering ligands, such as saccharides, vitamins, bisphosphonate, antibodies, peptides, and aptamers, to the delivery vehicles [167,168]. For example, chemical conjugation of folic acid, a vitamin, to bacteriophage pRNA-based delivery system enables specific recognition of folate receptors that are overexpressed on the surface of cancer cells but are barely detectable on normal tissues [169]. Zhang et al. successfully targeted miR-145 to prostate cancer cells through the conjugation of polyarginine peptide (R11), a cell permeable peptide, to a branched PEI containing disulfide linkages [170]. Decoration of nanoparticles with galactose and glycyrrhetinic acid moieties significantly improved the efficiency and specificity of active targeting to the liver [171].

Several groups are investigating new avenues to develop unconventional delivery methods. EnGeneIC Ltd. (Sydney, Australia) developed an antibody-coated bacterially derived minicell (400 nm) delivery system that can package and deliver chemotherapeutics to targeted cells [172]. Later, several groups adapted this bacteria-based technology deliver miRNAs in pre-clinical [173,174] and clinical trials (dubbed TargomiRs; see Table 2) for cancer treatment. A growing body of work is now focusing on developing 3D biomaterial scaffolds, e.g., hydrogels, and electrospun fibers, for miRNA delivery (reviewed in [59]). A limited number of recent studies have suggested that dietary, particularly plant-based, delivery of miRNAs could provide an effective, noninvasive, and inexpensive treatment regime for some human diseases [175]. With the recent advances in next-generation sequencing technologies and bioinformatic tools, the inventory of novel miRNAs associated with human health and disease will continue to surge over the next decade. The development of new delivery technologies and their evaluation in animal models will be a promising research area. Additionally, future studies should focus on the characterization of disease-specific markers on target tissues and explore new targeting ligands for improving miRNA therapeutic efficacy.

## Figures and Tables

**Table 1:** Selected list of miRNA-based therapeutics.

Delivery System	miRNA	Target Disease	Cellular Targets	Reference
**Viral Systems**				
Lentiviral-Based Delivery Systems				
Lentiviral	miR-133b	Spinal cord regeneration	RhoA, Xylt1, Epha7, P2X, P2RX4	[37]
Lentiviral	let-7	Non-small-cell lung cancer (NSCLC)	*RAS*, *MYC*, *HMGA2*, *CDC25A*, *CDK6,* cyclin-D2	[38]
Adeno-associated virus (AAV) serotype 3	miR-26a miR-122	Liver tumor	PIK3C2α/Akt/HIF-1α/VEGFA Bcl-2, Bcl-w, Bcl-xl, and Mcl-1	[39]
AAV serotype 9	miR-298	Spinal and bulbar muscular atrophy	Androgen receptor	[40]
AAV serotype 5	mi*ATXN3*	Spinocerebellar ataxia type 3 (SCA3)	ATXN3	[41]
**Non-Viral Systems**				
Lipid-Based Delivery Systems				
Lipid nanoparticle	ds-miR-634	Pancreatic cancer	OPA1, TFAM, APIP, XIAP, and BIRC5, NRF2, LAMP2	[42]
Neutral liposome	miR-34a	Lung cancer	BCL-2, c-Met, KRAS	[43]
Cationic liposome	anti-miR-712	Atherosclerosis	TIMP3, MMPs, ADAMs	[44]
Cationic liposome	miR-143 miR-145	Colorectal carcinoma	ERK5, K-ras, CHEK2 MYCN, FOS, YES, FLI, cyclin D2, cyclin CDK3, MAP3K3, MAPK4K4	[44]
Cationic liposome	miR-7	Lung cancer	IRS-1, RAF-1, EGFR	[45]
Cationic liposome	miR-29b	Lung cancer	CDK6, DNMT3B, MCL1	[46]
Ionizable liposome	miR-200c	Lung cancer	PRDX2, SESN1, GAPB/Nrf2	[47]
Ionizable cationic lipid nanoparticles	miR-199b-5p	Colon, breast, prostate, glioblastoma, medulloblastoma cancer	Hes-1	[48]
Polymeric Delivery Systems				
Polyethyleneimines (PEI)	miR-145 miR-33a	Colon carcinoma	c-Myc, ERK5	[49]
PEI-PEG	miR-34a	Hepatocellular carcinoma	SNAI1	[50]
PACE polymer	anti-miR-21	Glioblastoma	PTEN	[51]
Polymer micelle	anti-miR-21	Glioma	PTEN	[52]
Inorganic Compound-Based Delivery Systems				
Carbonate apatite	miR-29b miR-4689	Colorectal cancers	BCL-2, MCL1 KRAS, AKT1	[53,54]
Exosome-Based Delivery Systems				
Exosomes	miR-199a-3p	Ovarian cancer	c-Met, mTOR, IKKβ, and CD44	[55]
Exosome-GE11 peptide	let-7	Breast cancer	HMGA2	[56]
Exosome	miR-122	Hepatocellular carcinoma	ADAM10, IGF1R, CCNG1	[57]
Exosome	miR-145	Lung cancer	CDH2	[58]

**Table 2:** Selected list of miRNA-based clinical trials.

Developmental Drug	miRNA	Disease	Phase	Agency/Company
Miravirsen	anti-miR-122	Hepatitis C virus infection	II	Santaris Pharma
RG-101	anti-miR-122	Hepatitis C virus infection	II	Regulus Therapeutics
MRX34	miR-34	Cancer treatment	I	Mirna Therapeutics
RG-012	anti-miR-21	Alport syndrome	I	Regulus Therapeutics
MesomiR-1	miR-16	Malignant pleural mesothelioma or NSCLC	I	EnGeneIC/Asbestos Diseases Research Institute
MRG-201	miR-29	Scleroderma	I	miRagen Therapeutics
MRG-106	anti-miR-155	Cutaneous T cell lymphoma	I	miRagen Therapeutics
RG-125	anti-miR-103/107	Non-alcoholic steatohepatitis	I	Regulus Therapeutics
RG-125 (AZD4076)	anti-miR-103/107	Type 2 diabetes	I	AstraZeneca
RGLS4326	anti-miR-17	Polycystic kidney disease (PKD)	I	Regulus Therapeutics
Cobomarsen (MRG-106)	anti-miR-155	Cutaneous T-cell lymphoma (CTCL)	I	miRagen therapeutics
MRG-110	anti-miR-92a	Ischemia	I	miRagen therapeutics
TargomiRs	miR-16	Malignant pleural mesothelioma	I	Asbestos Diseases Research Foundation
MRG-106	anti-miR-155	Cutaneous T cell lymphoma and mycosis fungoides	I	miRagen Therapeutics
MRG-107	anti-miR-155	Amyotrophic lateral sclerosis (ALS)	Entering clinical trial	miRagen therapeutics

## Data Availability

Not applicable.

## References

[1] He L., Hannon G.J. (2004). MicroRNAs: Small RNAs with a big role in gene regulation. Nat. Rev. Genet..

[2] Lai E.C. (2002). Micro RNAs are complementary to 3′ UTR sequence motifs that mediate negative post-transcriptional regulation. Nat. Genet..

[3] Lim L.P., Lau N.C., Garrett-Engele P., Grimson A., Schelter J.M., Castle J., Bartel D.P., Linsley P.S., Johnson J.M. (2005). Microarray analysis shows that some microRNAs downregulate large numbers of target mRNAs. Nature.

[4] Olsen P.H., Ambros V. (1999). The lin-4 regulatory RNA controls developmental timing in Caenorhabditis elegans by blocking LIN-14 protein synthesis after the initiation of translation. Dev. Biol..

[5] Hammond S.M. (2015). An overview of microRNAs. Adv. Drug Deliv. Rev..

[6] Kozomara A., Birgaoanu M., Griffiths-Jones S. (2019). miRBase: From microRNA sequences to function. Nucleic Acids Res..

[7] Labatut A.E., Mattheolabakis G. (2018). Non-viral based miR delivery and recent developments. Eur. J. Pharm. Biopharm..

[8] Wilson R.C., Doudna J.A. (2013). Molecular mechanisms of RNA interference. Annu. Rev. Biophys..

[9] Landgraf P., Rusu M., Sheridan R., Sewer A., Iovino N., Aravin A., Pfeffer S., Rice A., Kamphorst A.O., Landthaler M. (2007). A mammalian microRNA expression atlas based on small RNA library sequencing. Cell.

[10] Ludwig N., Leidinger P., Becker K., Backes C., Fehlmann T., Pallasch C., Rheinheimer S., Meder B., Stahler C., Meese E. (2016). Distribution of miRNA expression across human tissues. Nucleic Acids Res..

[11] Drury R.E., O’Connor D., Pollard A.J. (2017). The Clinical Application of MicroRNAs in Infectious Disease. Front. Immunol..

[12] Finotti A., Fabbri E., Lampronti I., Gasparello J., Borgatti M., Gambari R. (2019). MicroRNAs and Long Non-coding RNAs in Genetic Diseases. Mol. Diagn. Ther..

[13] Markopoulos G.S., Roupakia E., Tokamani M., Alabasi G., Sandaltzopoulos R., Marcu K.B., Kolettas E. (2018). Roles of NF-kappaB Signaling in the Regulation of miRNAs Impacting on Inflammation in Cancer. Biomedicines.

[14] McCoy C.E. (2017). miR-155 Dysregulation and Therapeutic Intervention in Multiple Sclerosis. Adv. Exp. Med. Biol..

[15] Miya Shaik M., Tamargo I.A., Abubakar M.B., Kamal M.A., Greig N.H., Gan S.H. (2018). The Role of microRNAs in Alzheimer’s Disease and Their Therapeutic Potentials. Genes.

[16] Strumidlo A., Skiba S., Scott R.J., Lubinski J. (2017). The potential role of miRNAs in therapy of breast and ovarian cancers associated with BRCA1 mutation. Hered Cancer Clin. Pract..

[17] Wojciechowska A., Braniewska A., Kozar-Kaminska K. (2017). MicroRNA in cardiovascular biology and disease. Adv. Clin. Exp. Med..

[18] Esau C.C., Monia B.P. (2007). Therapeutic potential for microRNAs. Adv. Drug Deliv. Rev..

[19] Li Z., Rana T.M. (2014). Therapeutic targeting of microRNAs: Current status and future challenges. Nat. Rev. Drug Discov..

[20] Bader A.G., Brown D., Winkler M. (2010). The promise of microRNA replacement therapy. Cancer Res..

[21] Banales J.M., Saez E., Uriz M., Sarvide S., Urribarri A.D., Splinter P., Tietz Bogert P.S., Bujanda L., Prieto J., Medina J.F. (2012). Up-regulation of microRNA 506 leads to decreased Cl-/HCO_3_- anion exchanger 2 expression in biliary epithelium of patients with primary biliary cirrhosis. Hepatology.

[22] Gambari R., Brognara E., Spandidos D.A., Fabbri E. (2016). Targeting oncomiRNAs and mimicking tumor suppressor miRNAs: New trends in the development of miRNA therapeutic strategies in oncology. Int. J. Oncol..

[23] Cheng C.J., Bahal R., Babar I.A., Pincus Z., Barrera F., Liu C., Svoronos A., Braddock D.T., Glazer P.M., Engelman D.M. (2015). MicroRNA silencing for cancer therapy targeted to the tumour microenvironment. Nature.

[24] Tang L., Chen H.Y., Hao N.B., Tang B., Guo H., Yong X., Dong H., Yang S.M. (2017). microRNA inhibitors: Natural and artificial sequestration of microRNA. Cancer Lett..

[25] Bravo V., Rosero S., Ricordi C., Pastori R.L. (2007). Instability of miRNA and cDNAs derivatives in RNA preparations. Biochem. Biophys. Res. Commun..

[26] Cho W.C. (2009). Role of miRNAs in lung cancer. Expert Rev. Mol. Diagn..

[27] Grimm D., Streetz K.L., Jopling C.L., Storm T.A., Pandey K., Davis C.R., Marion P., Salazar F., Kay M.A. (2006). Fatality in mice due to oversaturation of cellular microRNA/short hairpin RNA pathways. Nature.

[28] Pecot C.V., Calin G.A., Coleman R.L., Lopez-Berestein G., Sood A.K. (2011). RNA interference in the clinic: Challenges and future directions. Nat. Rev. Cancer.

[29] Wang H., Jiang Y., Peng H., Chen Y., Zhu P., Huang Y. (2015). Recent progress in microRNA delivery for cancer therapy by non-viral synthetic vectors. Adv. Drug Deliv. Rev..

[30] Crooke S.T., Graham M.J., Zuckerman J.E., Brooks D., Conklin B.S., Cummins L.L., Greig M.J., Guinosso C.J., Kornbrust D., Manoharan M. (1996). Pharmacokinetic properties of several novel oligonucleotide analogs in mice. J. Pharmacol. Exp. Ther..

[31] Van Rooij E., Kauppinen S. (2014). Development of microRNA therapeutics is coming of age. EMBO Mol. Med..

[32] Wahlestedt C., Salmi P., Good L., Kela J., Johnsson T., Hokfelt T., Broberger C., Porreca F., Lai J., Ren K. (2000). Potent and nontoxic antisense oligonucleotides containing locked nucleic acids. Proc. Natl. Acad. Sci. USA.

[33] Sun X., Guo Q., Wei W., Robertson S., Yuan Y., Luo X. (2019). Current Progress on MicroRNA-Based Gene Delivery in the Treatment of Osteoporosis and Osteoporotic Fracture. Int. J. Endocrinol..

[34] Chen Y., Gao D.Y., Huang L. (2015). In vivo delivery of miRNAs for cancer therapy: Challenges and strategies. Adv. Drug. Deliv. Rev..

[35] Forterre A., Komuro H., Aminova S., Harada M. (2020). A Comprehensive Review of Cancer MicroRNA Therapeutic Delivery Strategies. Cancers.

[36] Liu C.G., Song J., Zhang Y.Q., Wang P.C. (2014). MicroRNA-193b is a regulator of amyloid precursor protein in the blood and cerebrospinal fluid derived exosomal microRNA-193b is a biomarker of Alzheimer’s disease. Mol. Med. Rep..

[37] Theis T., Yoo M., Park C.S., Chen J., Kugler S., Gibbs K.M., Schachner M. (2017). Lentiviral delivery of miR-133b improves functional recovery after spinal cord injury in mice. Mol. Neurobiol..

[38] Trang P., Medina P.P., Wiggins J.F., Ruffino L., Kelnar K., Omotola M., Homer R., Brown D., Bader A.G., Weidhaas J.B. (2010). Regression of murine lung tumors by the let-7 microRNA. Oncogene.

[39] Yin L., Keeler G.D., Zhang Y., Hoffman B.E., Ling C., Qing K., Srivastava A. (2020). AAV3-miRNA vectors for growth suppression of human hepatocellular carcinoma cells in vitro and human liver tumors in a murine xenograft model in vivo. Gene. Ther..

[40] Pourshafie N., Lee P.R., Chen K.L., Harmison G.G., Bott L.C., Fischbeck K.H., Rinaldi C. (2018). Systemic delivery of microRNA using recombinant adeno-associated virus serotype 9 to treat neuromuscular diseases in rodents. J. Vis. Exp..

[41] Martier R., Sogorb-Gonzalez M., Stricker-Shaver J., Hubener-Schmid J., Keskin S., Klima J., Toonen L.J., Juhas S., Juhasova J., Ellederova Z. (2019). Development of an AAV-Based microRNA gene therapy to treat Machado-Joseph Disease. Mol. Ther. Methods Clin. Dev..

[42] Gokita K., Inoue J., Ishihara H., Kojima K., Inazawa J. (2020). Therapeutic potential of LNP-mediated delivery of miR-634 for cancer therapy. Mol. Ther. Nucleic Acids.

[43] Misso G., Di Martino M.T., De Rosa G., Farooqi A.A., Lombardi A., Campani V., Zarone M.R., Gulla A., Tagliaferri P., Tassone P. (2014). Mir-34: A new weapon against cancer?. Mol. Ther. Nucleic Acids.

[44] Kheirolomoom A., Kim C.W., Seo J.W., Kumar S., Son D.J., Gagnon M.K., Ingham E.S., Ferrara K.W., Jo H. (2015). Multifunctional nanoparticles facilitate molecular targeting and miRNA delivery to inhibit atherosclerosis in ApoE(-/-) mice. ACS Nano.

[45] Rai K., Takigawa N., Ito S., Kashihara H., Ichihara E., Yasuda T., Shimizu K., Tanimoto M., Kiura K. (2011). Liposomal delivery of microRNA-7-expressing plasmid overcomes epidermal growth factor receptor tyrosine kinase inhibitor-resistance in lung cancer cells. Mol. Cancer Ther..

[46] Wu Y., Crawford M., Mao Y., Lee R.J., Davis I.C., Elton T.S., Lee L.J., Nana-Sinkam S.P. (2013). Therapeutic Delivery of MicroRNA-29b by Cationic Lipoplexes for Lung Cancer. Mol. Ther. Nucleic Acids.

[47] Cortez M.A., Valdecanas D., Zhang X., Zhan Y., Bhardwaj V., Calin G.A., Komaki R., Giri D.K., Quini C.C., Wolfe T. (2014). Therapeutic delivery of miR-200c enhances radiosensitivity in lung cancer. Mol. Ther..

[48] De Antonellis P., Liguori L., Falanga A., Carotenuto M., Ferrucci V., Andolfo I., Marinaro F., Scognamiglio I., Virgilio A., De Rosa G. (2013). MicroRNA 199b-5p delivery through stable nucleic acid lipid particles (SNALPs) in tumorigenic cell lines. Naunyn Schmiedebergs Arch. Pharmacol..

[49] Ibrahim A.F., Weirauch U., Thomas M., Grunweller A., Hartmann R.K., Aigner A. (2011). MicroRNA replacement therapy for miR-145 and miR-33a is efficacious in a model of colon carcinoma. Cancer Res..

[50] Hu Q., Wang K., Sun X., Li Y., Fu Q., Liang T., Tang G. (2016). A redox-sensitive, oligopeptide-guided, self-assembling, and efficiency-enhanced (ROSE) system for functional delivery of microRNA therapeutics for treatment of hepatocellular carcinoma. Biomaterials.

[51] Seo Y.E., Suh H.W., Bahal R., Josowitz A., Zhang J., Song E., Cui J., Noorbakhsh S., Jackson C., Bu T. (2019). Nanoparticle-mediated intratumoral inhibition of miR-21 for improved survival in glioblastoma. Biomaterials.

[52] Qian X., Long L., Shi Z., Liu C., Qiu M., Sheng J., Pu P., Yuan X., Ren Y., Kang C. (2014). Star-branched amphiphilic PLA-b-PDMAEMA copolymers for co-delivery of miR-21 inhibitor and doxorubicin to treat glioma. Biomaterials.

[53] Hiraki M., Nishimura J., Takahashi H., Wu X., Takahashi Y., Miyo M., Nishida N., Uemura M., Hata T., Takemasa I. (2015). Concurrent targeting of KRAS and AKT by miR-4689 is a novel treatment against mutant KRAS colorectal cancer. Mol. Ther. Nucleic Acids.

[54] Inoue A., Mizushima T., Wu X., Okuzaki D., Kambara N., Ishikawa S., Wang J., Qian Y., Hirose H., Yokoyama Y. (2018). A miR-29b byproduct sequence exhibits potent tumor-suppressive activities via inhibition of NF-kappaB signaling in KRAS-mutant colon cancer cells. Mol. Cancer Ther..

[55] Kobayashi M., Sawada K., Miyamoto M., Shimizu A., Yamamoto M., Kinose Y., Nakamura K., Kawano M., Kodama M., Hashimoto K. (2020). Exploring the potential of engineered exosomes as delivery systems for tumor-suppressor microRNA replacement therapy in ovarian cancer. Biochem. Biophys. Res. Commun..

[56] Ohno S., Takanashi M., Sudo K., Ueda S., Ishikawa A., Matsuyama N., Fujita K., Mizutani T., Ohgi T., Ochiya T. (2013). Systemically injected exosomes targeted to EGFR deliver antitumor microRNA to breast cancer cells. Mol. Ther..

[57] Lou G., Song X., Yang F., Wu S., Wang J., Chen Z., Liu Y. (2015). Exosomes derived from miR-122-modified adipose tissue-derived MSCs increase chemosensitivity of hepatocellular carcinoma. J. Hematol. Oncol..

[58] Vazquez-Rios A.J., Molina-Crespo A., Bouzo B.L., Lopez-Lopez R., Moreno-Bueno G., de la Fuente M. (2019). Exosome-mimetic nanoplatforms for targeted cancer drug delivery. J. Nanobiotechnol..

[59] Fu Y., Chen J., Huang Z. (2019). Recent progress in microRNA-based delivery systems for the treatment of human disease. ExRNA.

[60] Yang N. (2015). An overview of viral and nonviral delivery systems for microRNA. Int. J. Pharm. Investig..

[61] Pan Y., Zhang Y., Jia T., Zhang K., Li J., Wang L. (2012). Development of a microRNA delivery system based on bacteriophage MS2 virus-like particles. FEBS J..

[62] Sun Y., Sun Y., Zhao R. (2017). Establishment of microRNA delivery system by PP7 bacteriophage-like particles carrying cell-penetrating peptide. J. Biosci. Bioeng..

[63] Pages J.C., Bru T. (2004). Toolbox for retrovectorologists. J. Gene Med..

[64] Cavazzana-Calvo M., Hacein-Bey S., de Saint Basile G., Gross F., Yvon E., Nusbaum P., Selz F., Hue C., Certain S., Casanova J.L. (2000). Gene therapy of human severe combined immunodeficiency (SCID)-X1 disease. Science.

[65] Hacein-Bey-Abina S., Von Kalle C., Schmidt M., McCormack M.P., Wulffraat N., Leboulch P., Lim A., Osborne C.S., Pawliuk R., Morillon E. (2003). LMO2-associated clonal T cell proliferation in two patients after gene therapy for SCID-X1. Science.

[66] Ye D., Wang G., Liu Y., Huang W., Wu M., Zhu S., Jia W., Deng A.M., Liu H., Kang J. (2012). MiR-138 promotes induced pluripotent stem cell generation through the regulation of the p53 signaling. Stem Cells.

[67] Berkowitz R., Ilves H., Lin W.Y., Eckert K., Coward A., Tamaki S., Veres G., Plavec I. (2001). Construction and molecular analysis of gene transfer systems derived from bovine immunodeficiency virus. J. Virol..

[68] Leroux C., Cadore J.L., Montelaro R.C. (2004). Equine Infectious Anemia Virus (EIAV): What has HIV’s country cousin got to tell us?. Vet. Res..

[69] Mangeot P.E., Negre D., Dubois B., Winter A.J., Leissner P., Mehtali M., Kaiserlian D., Cosset F.L., Darlix J.L. (2000). Development of minimal lentivirus vectors derived from simian immunodeficiency virus (SIVmac251) and their use for gene transfer into human dendritic cells. J. Virol..

[70] Poeschla E., Gilbert J., Li X., Huang S., Ho A., Wong-Staal F. (1998). Identification of a human immunodeficiency virus type 2 (HIV-2) encapsidation determinant and transduction of nondividing human cells by HIV-2-based lentivirus vectors. J. Virol..

[71] Poeschla E.M., Wong-Staal F., Looney D.J. (1998). Efficient transduction of nondividing human cells by feline immunodeficiency virus lentiviral vectors. Nat. Med..

[72] Milone M.C., O’Doherty U. (2018). Clinical use of lentiviral vectors. Leukemia.

[73] Baum C., Dullmann J., Li Z., Fehse B., Meyer J., Williams D.A., von Kalle C. (2003). Side effects of retroviral gene transfer into hematopoietic stem cells. Blood.

[74] Von Kalle C., Fehse B., Layh-Schmitt G., Schmidt M., Kelly P., Baum C. (2004). Stem cell clonality and genotoxicity in hematopoietic cells: Gene activation side effects should be avoidable. Semin. Hematol..

[75] Laufs S., Guenechea G., Gonzalez-Murillo A., Zsuzsanna Nagy K., Luz Lozano M., del Val C., Jonnakuty S., Hotz-Wagenblatt A., Jens Zeller W., Bueren J.A. (2006). Lentiviral vector integration sites in human NOD/SCID repopulating cells. J. Gene Med..

[76] Montini E., Cesana D., Schmidt M., Sanvito F., Bartholomae C.C., Ranzani M., Benedicenti F., Sergi L.S., Ambrosi A., Ponzoni M. (2009). The genotoxic potential of retroviral vectors is strongly modulated by vector design and integration site selection in a mouse model of HSC gene therapy. J. Clin. Investig..

[77] Montini E., Cesana D., Schmidt M., Sanvito F., Ponzoni M., Bartholomae C., Sergi Sergi L., Benedicenti F., Ambrosi A., Di Serio C. (2006). Hematopoietic stem cell gene transfer in a tumor-prone mouse model uncovers low genotoxicity of lentiviral vector integration. Nat. Biotechnol..

[78] Kasar S., Salerno E., Yuan Y., Underbayev C., Vollenweider D., Laurindo M.F., Fernandes H., Bonci D., Addario A., Mazzella F. (2012). Systemic in vivo lentiviral delivery of miR-15a/16 reduces malignancy in the NZB *de novo* mouse model of chronic lymphocytic leukemia. Genes Immun..

[79] Liu Y., Lai L., Chen Q., Song Y., Xu S., Ma F., Wang X., Wang J., Yu H., Cao X. (2012). MicroRNA-494 is required for the accumulation and functions of tumor-expanded myeloid-derived suppressor cells via targeting of PTEN. J. Immunol..

[80] Daya S., Berns K.I. (2008). Gene therapy using adeno-associated virus vectors. Clin. Microbiol. Rev..

[81] Schultz B.R., Chamberlain J.S. (2008). Recombinant adeno-associated virus transduction and integration. Mol. Ther..

[82] Miyazaki Y., Adachi H., Katsuno M., Minamiyama M., Jiang Y.M., Huang Z., Doi H., Matsumoto S., Kondo N., Iida M. (2012). Viral delivery of miR-196a ameliorates the SBMA phenotype via the silencing of CELF2. Nat. Med..

[83] Tang X., Zhang H., Song Y., Zhou D., Wang J. (2016). Hemagglutinin-targeting artificial microRNAs expressed by adenovirus protect mice from different clades of H5N1 infection. Mol. Ther. Nucleic Acids.

[84] Vandenberghe L.H., Wilson J.M. (2007). AAV as an immunogen. Curr. Gene Ther..

[85] Wang B., Li J., Fu F.H., Chen C., Zhu X., Zhou L., Jiang X., Xiao X. (2008). Construction and analysis of compact muscle-specific promoters for AAV vectors. Gene Ther..

[86] Qiao C., Yuan Z., Li J., He B., Zheng H., Mayer C., Li J., Xiao X. (2011). Liver-specific microRNA-122 target sequences incorporated in AAV vectors efficiently inhibits transgene expression in the liver. Gene Ther..

[87] Tripp R.A., Mark Tompkins S. (2015). Antiviral effects of inhibiting host gene expression. Curr. Top. Microbiol. Immunol..

[88] Cooray S., Howe S.J., Thrasher A.J. (2012). Retrovirus and lentivirus vector design and methods of cell conditioning. Methods Enzymol..

[89] Vannucci L., Lai M., Chiuppesi F., Ceccherini-Nelli L., Pistello M. (2013). Viral vectors: A look back and ahead on gene transfer technology. New Microbiol..

[90] Pan Y., Jia T., Zhang Y., Zhang K., Zhang R., Li J., Wang L. (2012). MS2 VLP-based delivery of microRNA-146a inhibits autoantibody production in lupus-prone mice. Int. J. Nanomed..

[91] Wang G., Jia T., Xu X., Chang L., Zhang R., Fu Y., Li Y., Yang X., Zhang K., Lin G. (2016). Novel miR-122 delivery system based on MS2 virus like particle surface displaying cell-penetrating peptide TAT for hepatocellular carcinoma. Oncotarget.

[92] Yao Y., Jia T., Pan Y., Gou H., Li Y., Sun Y., Zhang R., Zhang K., Lin G., Xie J. (2015). Using a novel microRNA delivery system to inhibit osteoclastogenesis. Int. J. Mol. Sci..

[93] Ye X., Liu Z., Hemida M.G., Yang D. (2011). Targeted delivery of mutant tolerant anti-coxsackievirus artificial microRNAs using folate conjugated bacteriophage Phi29 pRNA. PLoS ONE.

[94] Guo P.X., Erickson S., Anderson D. (1987). A small viral RNA is required for in vitro packaging of bacteriophage phi 29 DNA. Science.

[95] Guo P. (2005). Bacterial virus phi29 DNA-packaging motor and its potential applications in gene therapy and nanotechnology. Methods Mol. Biol..

[96] Zhang Y., Wang Z., Gemeinhart R.A. (2013). Progress in microRNA delivery. J. Control. Release.

[97] Hsu S.H., Yu B., Wang X., Lu Y., Schmidt C.R., Lee R.J., Lee L.J., Jacob S.T., Ghoshal K. (2013). Cationic lipid nanoparticles for therapeutic delivery of siRNA and miRNA to murine liver tumor. Nanomedicine.

[98] Pedroso de Lima M.C., Simões S., Pires P., Faneca H., Düzgüneş N. (2001). Cationic lipid–DNA complexes in gene delivery: From biophysics to biological applications. Adv. Drug Deliv. Rev..

[99] Hara E.S., Ono M., Eguchi T., Kubota S., Pham H.T., Sonoyama W., Tajima S., Takigawa M., Calderwood S.K., Kuboki T. (2013). miRNA-720 controls stem cell phenotype, proliferation and differentiation of human dental pulp cells. PLoS ONE.

[100] Sluijter J.P., van Mil A., van Vliet P., Metz C.H., Liu J., Doevendans P.A., Goumans M.J. (2010). MicroRNA-1 and -499 regulate differentiation and proliferation in human-derived cardiomyocyte progenitor cells. Arterioscler. Thromb. Vasc. Biol..

[101] Zhao C., Sun G., Li S., Shi Y. (2009). A feedback regulatory loop involving microRNA-9 and nuclear receptor TLX in neural stem cell fate determination. Nat. Struct. Mol. Biol..

[102] Sundaram G.M., Common J.E., Gopal F.E., Srikanta S., Lakshman K., Lunny D.P., Lim T.C., Tanavde V., Lane E.B., Sampath P. (2013). ‘See-saw’ expression of microRNA-198 and FSTL1 from a single transcript in wound healing. Nature.

[103] Silva B.F., Majzoub R.N., Chan C.L., Li Y., Olsson U., Safinya C.R. (2014). PEGylated cationic liposome-DNA complexation in brine is pathway-dependent. Biochim. Biophys. Acta.

[104] Endo-Takahashi Y., Negishi Y., Nakamura A., Ukai S., Ooaku K., Oda Y., Sugimoto K., Moriyasu F., Takagi N., Suzuki R. (2014). Systemic delivery of miR-126 by miRNA-loaded bubble liposomes for the treatment of hindlimb ischemia. Sci. Rep..

[105] Shi S., Han L., Gong T., Zhang Z., Sun X. (2013). Systemic delivery of microRNA-34a for cancer stem cell therapy. Angew. Chem. Int. Ed. Engl..

[106] Piao L., Zhang M., Datta J., Xie X., Su T., Li H., Teknos T.N., Pan Q. (2012). Lipid-based nanoparticle delivery of pre-miR-107 inhibits the tumorigenicity of head and neck squamous cell carcinoma. Mol. Ther..

[107] Xu F., Liao J.Z., Xiang G.Y., Zhao P.X., Ye F., Zhao Q., He X.X. (2017). MiR-101 and doxorubicin codelivered by liposomes suppressing malignant properties of hepatocellular carcinoma. Cancer Med..

[108] Reddy T.L., Garikapati K.R., Reddy S.G., Reddy B.V., Yadav J.S., Bhadra U., Bhadra M.P. (2016). Simultaneous delivery of Paclitaxel and Bcl-2 siRNA via pH-Sensitive liposomal nanocarrier for the synergistic treatment of melanoma. Sci. Rep..

[109] Nishimura M., Jung E.J., Shah M.Y., Lu C., Spizzo R., Shimizu M., Han H.D., Ivan C., Rossi S., Zhang X. (2013). Therapeutic synergy between microRNA and siRNA in ovarian cancer treatment. Cancer Discov..

[110] Yang D., Sun Y., Hu L., Zheng H., Ji P., Pecot C.V., Zhao Y., Reynolds S., Cheng H., Rupaimoole R. (2013). Integrated analyses identify a master microRNA regulatory network for the mesenchymal subtype in serous ovarian cancer. Cancer Cell.

[111] Pecot C.V., Rupaimoole R., Yang D., Akbani R., Ivan C., Lu C., Wu S., Han H.D., Shah M.Y., Rodriguez-Aguayo C. (2013). Tumour angiogenesis regulation by the miR-200 family. Nat. Commun..

[112] Zylberberg C., Matosevic S. (2016). Pharmaceutical liposomal drug delivery: A review of new delivery systems and a look at the regulatory landscape. Drug Deliv..

[113] Wilner S.E., Levy M. (2016). Synthesis and Characterization of Aptamer-Targeted SNALPs for the Delivery of siRNA. Methods Mol. Biol..

[114] Hobel S., Aigner A. (2013). Polyethylenimines for siRNA and miRNA delivery in vivo. Wiley Interdiscip. Rev. Nanomed. Nanobiotechnol..

[115] Jager M., Schubert S., Ochrimenko S., Fischer D., Schubert U.S. (2012). Branched and linear poly(ethylene imine)-based conjugates: Synthetic modification, characterization, and application. Chem. Soc. Rev..

[116] Gao S., Tian H., Guo Y., Li Y., Guo Z., Zhu X., Chen X. (2015). miRNA oligonucleotide and sponge for miRNA-21 inhibition mediated by PEI-PLL in breast cancer therapy. Acta Biomater..

[117] Biray Avci C., Ozcan I., Balci T., Ozer O., Gunduz C. (2013). Design of polyethylene glycol-polyethylenimine nanocomplexes as non-viral carriers: Mir-150 delivery to chronic myeloid leukemia cells. Cell Biol. Int..

[118] Cheng C.J., Saltzman W.M. (2012). Polymer nanoparticle-mediated delivery of microRNA inhibition and alternative splicing. Mol. Pharm..

[119] Wang F., Zhang B., Zhou L., Shi Y., Li Z., Xia Y., Tian J. (2016). Imaging dendrimer-grafted graphene oxide mediated anti-miR-21 delivery with an activatable luciferase reporter. ACS Appl. Mater. Interfaces.

[120] Salzano G., Costa D.F., Sarisozen C., Luther E., Mattheolabakis G., Dhargalkar P.P., Torchilin V.P. (2016). Mixed nanosized polymeric micelles as promoter of doxorubicin and miRNA-34a co-delivery triggered by dual stimuli in tumor tissue. Small.

[121] Suh J.S., Lee J.Y., Choi Y.S., Chung C.P., Park Y.J. (2013). Peptide-mediated intracellular delivery of miRNA-29b for osteogenic stem cell differentiation. Biomaterials.

[122] Gao S., Chen J., Dong L., Ding Z., Yang Y.H., Zhang J. (2005). Targeting delivery of oligonucleotide and plasmid DNA to hepatocyte via galactosylated chitosan vector. Eur. J. Pharm. Biopharm..

[123] Gao S., Chen J., Xu X., Ding Z., Yang Y.H., Hua Z., Zhang J. (2003). Galactosylated low molecular weight chitosan as DNA carrier for hepatocyte-targeting. Int. J. Pharm..

[124] Martirosyan A., Olesen M.J., Howard K.A. (2014). Chitosan-based nanoparticles for mucosal delivery of RNAi therapeutics. Adv. Genet..

[125] Chen Y., Xianyu Y., Jiang X. (2017). Surface modification of gold nanoparticles with small molecules for biochemical analysis. Acc. Chem. Res..

[126] Schade A., Delyagina E., Scharfenberg D., Skorska A., Lux C., David R., Steinhoff G. (2013). Innovative strategy for microRNA delivery in human mesenchymal stem cells via magnetic nanoparticles. Int. J. Mol. Sci..

[127] Bitar A., Ahmad N.M., Fessi H., Elaissari A. (2012). Silica-based nanoparticles for biomedical applications. Drug Discov. Today.

[128] Jia C., Chen H., Wei M., Chen X., Zhang Y., Cao L., Yuan P., Wang F., Yang G., Ma J. (2017). Gold nanoparticle-based miR155 antagonist macrophage delivery restores the cardiac function in ovariectomized diabetic mouse model. Int. J. Nanomed..

[129] Ghosh R., Singh L.C., Shohet J.M., Gunaratne P.H. (2013). A gold nanoparticle platform for the delivery of functional microRNAs into cancer cells. Biomaterials.

[130] Li Y., Duo Y., Bi J., Zeng X., Mei L., Bao S., He L., Shan A., Zhang Y., Yu X. (2018). Targeted delivery of anti-miR-155 by functionalized mesoporous silica nanoparticles for colorectal cancer therapy. Int. J. Nanomed..

[131] Mamaeva V., Sahlgren C., Linden M. (2013). Mesoporous silica nanoparticles in medicine-recent advances. Adv. Drug Deliv. Rev..

[132] Sun S., Wang Y., Zhou R., Deng Z., Han Y., Han X., Tao W., Yang Z., Shi C., Hong D. (2017). Targeting and regulating of an oncogene via nanovector delivery of microRNA using patient-derived xenografts. Theranostics.

[133] Andaloussi S.E., Mager I., Breakefield X.O., Wood M.J. (2013). Extracellular vesicles: Biology and emerging therapeutic opportunities. Nat. Rev. Drug Discov..

[134] De Jong O.G., Kooijmans S.A.A., Murphy D.E., Jiang L., Evers M.J.W., Sluijter J.P.G., Vader P., Schiffelers R.M. (2019). Drug Delivery with Extracellular Vesicles: From Imagination to Innovation. Acc. Chem. Res..

[135] Ha D., Yang N., Nadithe V. (2016). Exosomes as therapeutic drug carriers and delivery vehicles across biological membranes: Current perspectives and future challenges. Acta Pharm. Sin. B.

[136] Simons M., Raposo G. (2009). Exosomes-vesicular carriers for intercellular communication. Curr. Opin. Cell Biol..

[137] Munir J., Yoon J.K., Ryu S. (2020). Therapeutic miRNA-Enriched Extracellular Vesicles: Current Approaches and Future Prospects. Cells.

[138] Jiang M., Wang H., Jin M., Yang X., Ji H., Jiang Y., Zhang H., Wu F., Wu G., Lai X. (2018). Exosomes from MiR-30d-5p-ADSCs Reverse Acute Ischemic Stroke-Induced, Autophagy-Mediated Brain Injury by Promoting M2 Microglial/Macrophage Polarization. Cell Physiol. Biochem..

[139] Li D., Huang S., Yin Z., Zhu J., Ge X., Han Z., Tan J., Zhang S., Zhao J., Chen F. (2019). Increases in miR-124-3p in Microglial Exosomes Confer Neuroprotective Effects by Targeting FIP200-Mediated Neuronal Autophagy Following Traumatic Brain Injury. Neurochem. Res..

[140] Luarte A., Henzi R., Fernandez A., Gaete D., Cisternas P., Pizarro M., Batiz L.F., Villalobos I., Masalleras M., Vergara R. (2020). Astrocyte-Derived Small Extracellular Vesicles Regulate Dendritic Complexity through miR-26a-5p Activity. Cells.

[141] Xin H., Li Y., Buller B., Katakowski M., Zhang Y., Wang X., Shang X., Zhang Z.G., Chopp M. (2012). Exosome-mediated transfer of miR-133b from multipotent mesenchymal stromal cells to neural cells contributes to neurite outgrowth. Stem Cells.

[142] Luo Q., Guo D., Liu G., Chen G., Hang M., Jin M. (2017). Exosomes from MiR-126-Overexpressing Adscs Are Therapeutic in Relieving Acute Myocardial Ischaemic Injury. Cell Physiol. Biochem..

[143] Song Y., Zhang C., Zhang J., Jiao Z., Dong N., Wang G., Wang Z., Wang L. (2019). Localized injection of miRNA-21-enriched extracellular vesicles effectively restores cardiac function after myocardial infarction. Theranostics.

[144] Wang N., Chen C., Yang D., Liao Q., Luo H., Wang X., Zhou F., Yang X., Yang J., Zeng C. (2017). Mesenchymal stem cells-derived extracellular vesicles, via miR-210, improve infarcted cardiac function by promotion of angiogenesis. Biochim. Biophys. Acta Mol. Basis Dis..

[145] Chen S., Tang Y., Liu Y., Zhang P., Lv L., Zhang X., Jia L., Zhou Y. (2019). Exosomes derived from miR-375-overexpressing human adipose mesenchymal stem cells promote bone regeneration. Cell Prolif..

[146] Li X., Liu L.L., Yao J.L., Wang K., Ai H. (2019). Human Umbilical Cord Mesenchymal Stem Cell-Derived Extracellular Vesicles Inhibit Endometrial Cancer Cell Proliferation and Migration through Delivery of Exogenous miR-302a. Stem Cells Int..

[147] Wang F., Li L., Piontek K., Sakaguchi M., Selaru F.M. (2018). Exosome miR-335 as a novel therapeutic strategy in hepatocellular carcinoma. Hepatology.

[148] Morel L., Regan M., Higashimori H., Ng S.K., Esau C., Vidensky S., Rothstein J., Yang Y. (2013). Neuronal exosomal miRNA-dependent translational regulation of astroglial glutamate transporter GLT1. J. Biol. Chem..

[149] Zhou Y., Li P., Goodwin A.J., Cook J.A., Halushka P.V., Chang E., Fan H. (2018). Exosomes from Endothelial Progenitor Cells Improve the Outcome of a Murine Model of Sepsis. Mol. Ther..

[150] Wen D., Peng Y., Liu D., Weizmann Y., Mahato R.I. (2016). Mesenchymal stem cell and derived exosome as small RNA carrier and Immunomodulator to improve islet transplantation. J. Control. Release.

[151] Ortega A., Martinez-Arroyo O., Forner M.J., Cortes R. (2020). Exosomes as Drug Delivery Systems: Endogenous Nanovehicles for Treatment of Systemic Lupus Erythematosus. Pharmaceutics.

[152] Xu H., Jia S., Xu H. (2019). Potential therapeutic applications of exosomes in different autoimmune diseases. Clin. Immunol..

[153] Lee J.Y., Park J.K., Lee E.Y., Lee E.B., Song Y.W. (2020). Correction to: Circulating exosomes from patients with systemic lupus erythematosus induce an proinflammatory immune response. Arthritis Res. Ther..

[154] Zhang H., Bai M., Deng T., Liu R., Wang X., Qu Y., Duan J., Zhang L., Ning T., Ge S. (2016). Cell-derived microvesicles mediate the delivery of miR-29a/c to suppress angiogenesis in gastric carcinoma. Cancer Lett..

[155] Zernecke A., Bidzhekov K., Noels H., Shagdarsuren E., Gan L., Denecke B., Hristov M., Koppel T., Jahantigh M.N., Lutgens E. (2009). Delivery of microRNA-126 by apoptotic bodies induces CXCL12-dependent vascular protection. Sci. Signal.

[156] Gasparello J., Lomazzi M., Papi C., D’Aversa E., Sansone F., Casnati A., Donofrio G., Gambari R., Finotti A. (2019). Efficient delivery of microRNA and antimiRNA molecules using an argininocalix[4]arene macrocycle. Mol. Ther. Nucleic Acids.

[157] Gasparello J., Manicardi A., Casnati A., Corradini R., Gambari R., Finotti A., Sansone F. (2019). Efficient cell penetration and delivery of peptide nucleic acids by an argininocalix[4]arene. Sci. Rep..

[158] Soto-Sanchez C., Martinez-Navarrete G., Humphreys L., Puras G., Zarate J., Pedraz J.L., Fernandez E. (2015). Enduring high-efficiency in vivo transfection of neurons with non-viral magnetoparticles in the rat visual cortex for optogenetic applications. Nanomedicine.

[159] Titze de Almeida S.S., Horst C.H., Soto-Sanchez C., Fernandez E., Titze de Almeida R. (2018). Delivery of miRNA-targeted oligonucleotides in the rat striatum by magnetofection with neuromag((R)). Molecules.

[160] Zhang Q., Ding F., Liu X., Shen J., Su Y., Qian J., Zhu X., Zhang C. (2020). Nanobody-guided targeted delivery of microRNA via nucleic acid nanogel to inhibit the tumor growth. J. Control. Release.

[161] Qian H., Tay C.Y., Setyawati M.I., Chia S.L., Lee D.S., Leong D.T. (2017). Protecting microRNAs from RNase degradation with steric DNA nanostructures. Chem. Sci..

[162] Nahar S., Nayak A.K., Ghosh A., Subudhi U., Maiti S. (2017). Enhanced and synergistic downregulation of oncogenic miRNAs by self-assembled branched DNA. Nanoscale.

[163] Ban E., Kwon T.H., Kim A. (2019). Delivery of therapeutic miRNA using polymer-based formulation. Drug Deliv. Transl. Res..

[164] Chakraborty C., Sharma A.R., Sharma G., Doss C.G.P., Lee S.S. (2017). Therapeutic miRNA and siRNA: Moving from bench to clinic as next generation medicine. Mol. Ther. Nucleic Acids.

[165] Bikram M., Lee M., Chang C.W., Janat-Amsbury M.M., Kern S.E., Kim S.W. (2005). Long-circulating DNA-complexed biodegradable multiblock copolymers for gene delivery: Degradation profiles and evidence of dysopsonization. J. Control. Release.

[166] Bertucci A., Prasetyanto E.A., Septiadi D., Manicardi A., Brognara E., Gambari R., Corradini R., De Cola L. (2015). Combined delivery of Temozolomide and anti-miR221 PNA using mesoporous silica nanoparticles induces apoptosis in resistant glioma cells. Small.

[167] Huang X., Wu W., Yang W., Qing X., Shao Z. (2020). Surface engineering of nanoparticles with ligands for targeted delivery to osteosarcoma. Colloids Surf. B Biointerfaces.

[168] Li M., Zhang W., Wang B., Gao Y., Song Z., Zheng Q.C. (2016). Ligand-based targeted therapy: A novel strategy for hepatocellular carcinoma. Int. J. Nanomed..

[169] Lee T.J., Yoo J.Y., Shu D., Li H., Zhang J., Yu J.G., Jaime-Ramirez A.C., Acunzo M., Romano G., Cui R. (2017). RNA nanoparticle-based targeted therapy for glioblastoma through inhibition of oncogenic miR-21. Mol. Ther..

[170] Zhang T., Xue X., He D., Hsieh J.T. (2015). A prostate cancer-targeted polyarginine-disulfide linked PEI nanocarrier for delivery of microRNA. Cancer Lett..

[171] Chen H., Li M., Wan T., Zheng Q., Cheng M., Huang S., Wang Y. (2012). Design and synthesis of dual-ligand modified chitosan as a liver targeting vector. J. Mater. Sci. Mater. Med..

[172] MacDiarmid J.A., Mugridge N.B., Weiss J.C., Phillips L., Burn A.L., Paulin R.P., Haasdyk J.E., Dickson K.A., Brahmbhatt V.N., Pattison S.T. (2007). Bacterially derived 400 nm particles for encapsulation and cancer cell targeting of chemotherapeutics. Cancer Cell.

[173] Glover A.R., Zhao J.T., Gill A.J., Weiss J., Mugridge N., Kim E., Feeney A.L., Ip J.C., Reid G., Clarke S. (2015). MicroRNA-7 as a tumor suppressor and novel therapeutic for adrenocortical carcinoma. Oncotarget.

[174] Williams M., Kirschner M.B., Cheng Y.Y., Hanh J., Weiss J., Mugridge N., Wright C.M., Linton A., Kao S.C., Edelman J.J. (2015). miR-193a-3p is a potential tumor suppressor in malignant pleural mesothelioma. Oncotarget.

[175] Hirschi K.D., Pruss G.J., Vance V. (2015). Dietary delivery: A new avenue for microRNA therapeutics?. Trends Biotechnol..

